# Improving the oral health of older people in care homes (TOPIC): a protocol for a feasibility study

**DOI:** 10.1186/s40814-021-00872-6

**Published:** 2021-07-02

**Authors:** Georgios Tsakos, Paul R. Brocklehurst, Sinead Watson, Anna Verey, Nia Goulden, Alison Jenkins, Zoe Hoare, Kirstie Pye, Rebecca R. Wassall, Andrea Sherriff, Anja Heilmann, Ciaran O’Neill, Craig J. Smith, Joe Langley, Renato Venturelli, Peter Cairns, Nat Lievesley, Richard G. Watt, Frank Kee, Gerald McKenna

**Affiliations:** 1 Department of Epidemiology and Public Health, University College London, London, UK; 2 NWORTH Clinical Trials Unit, Bangor University, Bangor, UK; 3 Centre for Public Health, School of Medicine, Dentistry and Biomedical Sciences, Queen’s University Belfast, Belfast, UK; 4 School of Dental Sciences, Newcastle University, Newcastle, UK; 5 Dental School, University of Glasgow, Glasgow, UK; 6 Division of Cardiovascular Sciences, Lydia Becker Institute of Immunology and Inflammation, University of Manchester, Manchester, UK; 7 Manchester Centre for Clinical Neurosciences, Manchester Academic Health Science Centre, Salford Royal Foundation NHS Trust, Salford, UK; 8 Art and Design Research Centre, Sheffield Hallam University, Sheffield, UK; 9 BELONG PPI Group, Centre for Public Health, School of Medicine, Dentistry and Biomedical Sciences, Queen’s University, Belfast, UK; 10 Centre for Policy on Ageing, London, UK

**Keywords:** Care homes, Cluster randomised controlled trial, Complex intervention, Feasibility study, Older adults, Oral health

## Abstract

**Background:**

Evidence for interventions promoting oral health amongst care home residents is weak. The National Institute for Health and Care Excellence (NICE) guideline NG48 aims to maintain and improve the oral health of care home residents. A co-design process that worked with residents and care home staff to understand how the NG48 guideline could be best implemented in practice has been undertaken to refine a complex intervention. The aim of this study is to assess the feasibility of the intervention to inform a future larger scale definitive trial.

**Methods:**

This is a protocol for a pragmatic cluster randomised controlled trial with a 12-month follow-up that will be undertaken in 12 care homes across two sites (six in London, six in Northern Ireland). Care homes randomised to the intervention arm (n = 6) will receive the complex intervention based on the NG48 guideline, whilst care homes randomised to the control arm (n = 6) will continue with routine practice. The intervention will include a training package for care home staff to promote knowledge and skills in oral health promotion, the use of the Oral Health Assessment Tool on residents by trained care home staff, and a ‘support worker assisted’ daily tooth-brushing regime with toothpaste containing 1500 ppm fluoride. An average of ten residents, aged 65 years or over who have at least one natural tooth, will be recruited in each care home resulting in a recruited sample of 120 participants. Assessments will be undertaken at baseline, 6 months and 12 months, and will include a dental examination and questionnaires on general health and oral health administered by a research assistant. A parallel process evaluation involving semi-structured interviews will be undertaken to explore how the intervention could be embedded in standard practice. Rates of recruitment and retention, and intervention fidelity will also be recorded. A cost-consequence model will determine the relevance of different outcome measures in the decision-making context.

**Discussion:**

The study will provide valuable information for trialists, policymakers, clinicians and care home staff on the feasibility and associated costs of oral health promotion in UK care homes.

**Trial registration:**

ISRCTN10276613. Registered on 17th April 2020. http://www.isrctn.com/ISRCTN10276613.

## Introduction

### Background and rationale

Poor oral health is an increasingly common problem for older adults (defined as those over 65 years of age). According to the 2009 Adult Dental Health Survey undertaken in England, Wales and Northern Ireland, approximately 40% of the 75-84 age group and 33% of the 85+ age group had dental caries, whilst periodontal disease affected 69% of those over 65 years of age [1]. Oral conditions impact on the quality of life of older adults [2, 3] and their general health and diet [4, 5]. Access to domiciliary services is difficult, particularly for care home residents and hospital admission for dental problems can be distressing and costly [6, 7]. Income-related inequality in oral health of older adults is a major issue [8, 9]; therefore, effective prevention of oral diseases is paramount.

Approximately 400,000 older people live in care homes in the UK [10]. A care home is a broad term that refers to both residential and nursing care homes. About half of all care home residents have their own natural teeth [11] but their oral health is much worse than their peers living in the community (e.g. caries prevalence was 73% vs. 40%) [12]. Good daily oral hygiene is essential for oral health and the maintenance of complex dental restorations that are common amongst older adults. With increasing age, the ability to care for oral health (including dental restorations) can deteriorate and poly-pharmacy can lead to dry mouth [13]. Furthermore, diets can become rich in sugars [13]; especially in those who have a diminished appetite and rely on sugar to improve taste as well as provide additional calories to manage or prevent malnutrition and frailty. All these factors increase the risk of oral disease and directly impact on comorbidities, which in turn can worsen oral health.

Strategies for this population aim to prevent disease and to reduce pain and co-morbidity [14]. However, a Public Health England (PHE) survey showed oral healthcare provision and service in care homes to be poor [15]. A Priority Setting Partnership (PSP) exercise undertaken with four stakeholder groups, including service users, carers, third sector organisations and specialists such as those with specialist knowledge in Dental Public Health, Dental Health Commissioners and Geriatricians, found that maintaining function, dignity and the fear of losing the ability to look after their own teeth were key issues amongst older adults [16]. The World Health Organisation has focused on healthy ageing and prioritised the design of health and long-term care systems that are fit for ageing populations [17]. However, the evidence for interventions on promoting oral health amongst care home residents is weak [18]; no relevant systematic reviews have been published to date. There is uncertainty about effect size estimates, recruitment and retention of participants, intervention fidelity and appropriate outcome measures. This makes the design of a full trial problematic at this stage.

The National Institute for Health and Care Excellence (NICE) issued guideline NG48 [19], which aims to maintain and improve the oral health of care home residents. The aim of this multi-centre cluster randomised controlled trial (RCT) is to determine the feasibility of a complex intervention based on the NICE guideline for the oral health of older people in care homes. A parallel process evaluation will also be conducted, and a cost-consequence model developed to help plan for a definitive trial.

### Objectives

1. Determine the feasibility of undertaking a definitive trial to evaluate the complex intervention to promote oral health. To determine the following:
  - Proportion of care homes that agree to participate;
  - Number of residents that are eligible and able to consent;
  - Proportion of eligible residents that agree to participate;
  - Proportion of participating residents that receive the intervention per the protocol;
  - Proportion of care homes and residents that remain in the study;
  - Proportion of completed measures used in the study (at least 75% completion rate required): (i) oral health assessments, (ii) quality of life questionnaires, (iii) clinical measurement records, (iv) oral symptoms checklist diaries; and
  - Impact on recruitment of varying the 6-Cognitive Impairment Tool (6-CIT) [20] screening tool threshold.
2. Undertake a parallel process evaluation to explore how the intervention could be embedded in standard practice guided by Pfadenhauer et al.’s framework [21] to maximise pathways to impact. Semi-structured interviews will be conducted with the following:

- Managers and staff to assess the intervention’s feasibility and sustainability;
- Residents to explore the intervention’s acceptability; and
- Managers and residents that refused participation to explore their reasoning.

3. Develop a cost-consequence model to determine the relevance and relative importance of the different outcome measures in the decision-making context.

### Trial design

**Fig. 1:**
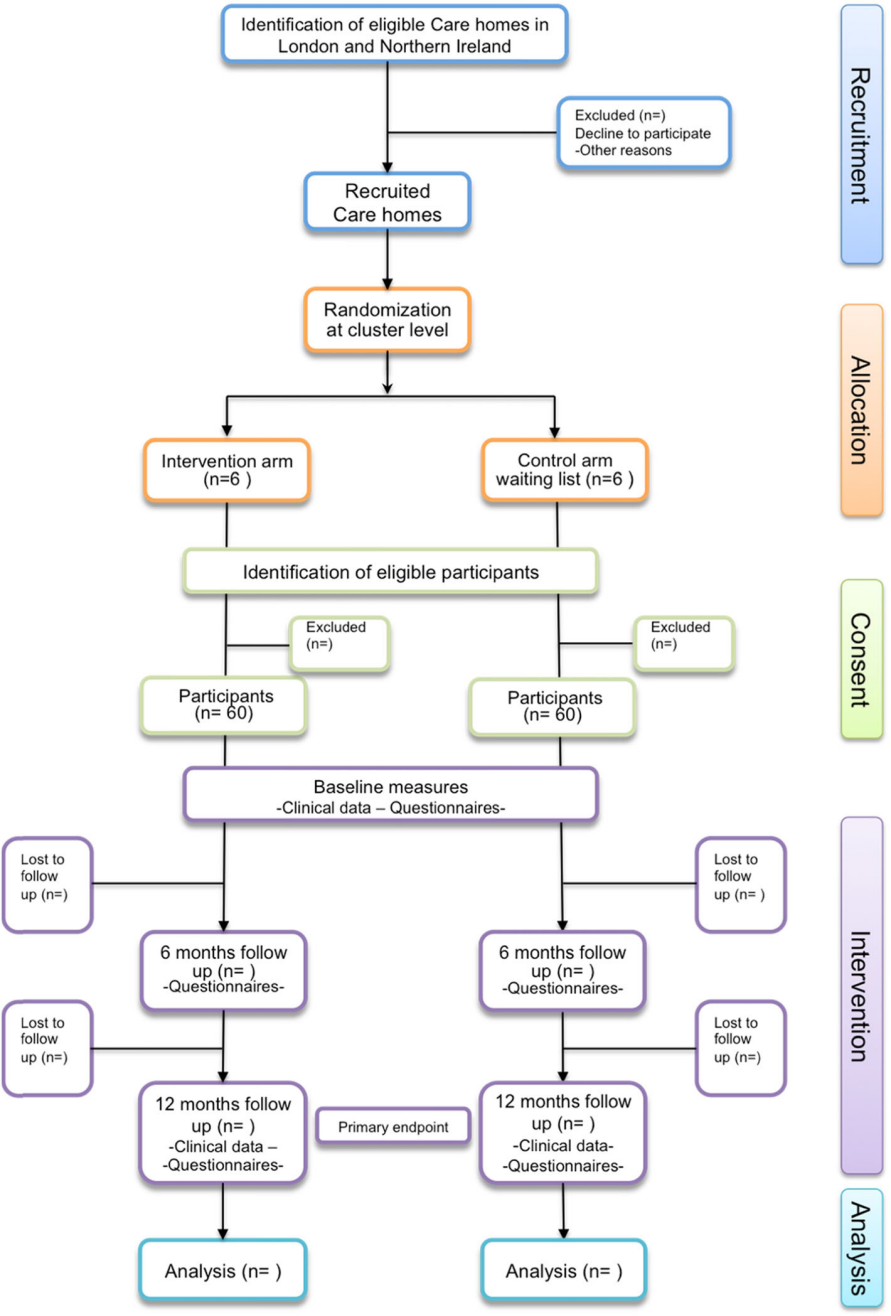
Flowchart of recruitment, allocation, consent, intervention and analysis

This is a pragmatic feasibility study to determine the feasibility of a multi-centre cluster randomised controlled trial of a complex intervention based on a recent NICE guideline for the oral health of older people in care homes [19]. Figure 1 provides an overview of the feasibility study’s design.

## Methods: Participants, interventions and outcomes

### Study setting

This feasibility study will be undertaken in approximately 12 privately owned care homes across two sites (North London and Northern Ireland) with recruitment on both arms of the study in each site. Further information about recruitment and randomisation is presented in the relevant sections below.

### Eligibility criteria

Care homes (cluster level) will be eligible to participate if they have a minimum of 20 residents (as approximately half are expected to be edentate). They will not be eligible to participate if they only have high-dependency units or provide end-of-life care.

Care home residents (individual level) will be eligible to participate if they meet the following inclusion criteria and have the capacity to provide consent:

1. Aged 65 years and over
2. Dentate or partially dentate
3. Full-time resident in care home

Care home residents will not be eligible to participate if they:

1. Are receiving end-of-life or palliative care
2. Have severe cognitive impairment (6-CIT score of 10 or higher)
3. Are currently taking part in another oral health intervention study
4. Do not have a working level of oral English

### Who will take informed consent?

For the two eligibility tests (6-CIT and brief dental check), written informed consent will be obtained from care home residents by a research assistant. For the feasibility study, written informed consent will be obtained from eligible care home residents by a research assistant or dental examiner. A research assistant will also take written informed consent for the interviews undertaken as part of the process evaluation and cost-consequence model. Written informed consent will be taken at least 48 hours after informing the participants about the study.

### Additional consent provisions for collection and use of participant data and biological specimens

Not applicable as no biological specimens will be collected as part of this trial.

## Interventions

### Explanation for the choice of comparators

Participants on the control arm will receive routine practice. The results from a PHE survey [15] and a PSP on the oral health of older adults [16] suggest that this practice is likely to be heterogeneous and include intermittent tooth brushing with toothpaste by the residents, who usually rely on support from the care home staff for their oral hygiene. This has been confirmed by a review of existing literature on oral health practices in care homes [22], as well as preliminary qualitative research findings from a recent project carried out in care homes in North London (unpublished). The process evaluation will enable the research team to gather information about current practice in care homes assigned to the control arm. Furthermore, collecting information on the control group’s willingness to be randomised to the control arm, as well as follow-up and response rates will help inform and plan for a future larger scale definitive trial.

### Intervention description

The description of the intervention below has been reported in accordance with the TIDieR (Template for Intervention Description and Replication) guidelines [23]. The intervention group will receive a complex intervention that fits within the Medical Research Council framework [24, 25] and is based on the recommendations of the NICE guideline (NG48) on ‘Oral health for adults in care homes’ [19]. The aim of the NG48 guideline is to maintain and improve the oral health of adults in care homes and ensure their timely access to dental treatment. The NG48 guideline is provided for the use of care home managers and care staff providing daily personal care to residents, community dental services and others. The guideline includes seven recommendations that cover oral health, including dental health and daily mouth care. The complex intervention will focus on three key recommendations that relate to the improvement of oral health without focusing on the provision of dental treatment: (1) care staff knowledge and skills, (2) oral health assessment and mouth care plans and (3) daily mouth care.

Intervention materials (Oral Health Assessment Tool (OHAT) [26], ‘Personal Oral Care Plan’, ‘Tips and tricks’ and ‘Weekly Oral Hygiene Record’) have been developed using a co-design process that worked with residents and care home staff to understand how the NG48 guideline could be best implemented in practice (method not described in this paper). This ensured that the materials were grounded in the experience of the older persons residing in care homes and those that provide their care. The aforementioned intervention materials are also supported by a care home staff training video to create a package of NG48-informed measures to promote knowledge and skills in oral health promotion, amongst care home staff. The training package had again been tested and refined by working with care home staff to promote fidelity. To further facilitate training and compliance, a dedicated web platform will be made available.

This complex intervention will include the following:

1. A care home staff training package (containing a training video and hard copy training manuals and laminated reference guides, as well as online training through a dedicated website as described above) to facilitate appropriate knowledge and skills to implement oral health promotion activities. Care home staff will be required to undertake formal training to support the residents’ oral health prior to the residents completing the baseline assessments. The training will be overseen by the care home manager and the research team, and will be added to the log of mandatory training. Following the initial training prior to baseline assessments, care home staff will have access to the training package online at anytime during the study. The turnover rate of employed staff working in the UK care sector is high; it is therefore probable that a change in care staff involved in the study will occur. As a result of this, the training package will also be part of the induction training for new staff members.
2. Administration by trained care home staff of OHAT, a brief and practical assessment of the resident’s oral health needs that is reviewed and updated over time. Administration will take place immediately prior to the initial dental assessment at baseline and at the 12-month follow-up visit. Care home staff will also be asked to complete a ‘Personal Oral Care Plan’ for each resident following completion of the OHAT, and will be asked to update it after reassessment of the OHAT or after any dental visit.
3. A ‘support worker assisted’ daily tooth-brushing regime with toothpaste containing 1500 ppm fluoride (provided by the study). This will involve care home staff brushing the teeth of residents who have problems with their oral self-care or providing assistance to those residents who are able to brush their own teeth. This may include reminders to brush their teeth as well as guidance on brushing appropriately. Tooth brushing will be undertaken twice daily, once in the morning and once in the evening. To facilitate this, a guide on how to deliver oral care will be provided in the form of a poster and ‘Tips and Tricks’ cards. These ‘Tips and Tricks’ cards are relevant to residents who can brush their own teeth, who require assistance or who might refuse care. Care home staff will be asked to record daily tooth brushing on the ‘Weekly Oral Hygiene Record’ along with any ‘Tips or Tricks’ they used. The record will be used to assess fidelity of the intervention implementation.

### Criteria for discontinuing or modifying allocated interventions

It is not expected that the oral health intervention described above will cause any adverse effects. Participants and care homes are free to withdraw from the study at any point in time. Given the co-design process undertaken prior to the feasibility study, there will be no modification of allocated treatments. However, a parallel process evaluation guided by Pfadenhauer et al.’s framework [21] will be undertaken to refine the intervention ahead of a definitive trial.

### Strategies to improve adherence to interventions

There will be no additional strategies to improve the adherence to the intervention. Adherence to the intervention will be monitored by collecting information on completion rates (fully, partially or not completed) for the following: (1) OHAT administered by trained care staff to participants, (2) Personal Care Plan for participants, (3) Weekly Oral Hygiene Record of participants, and (4) Care Staff Completed Training.

### Relevant concomitant care permitted or prohibited during the trial

In order to be pragmatic, the intervention will be delivered alongside any oral care practices currently in place in the care homes and being provided to residents. Care homes will not be asked to cease any practices that they are currently undertaking on either control or intervention arm. Existing care home practices will be recorded and reported as a part of the feasibility study.

### Provisions for post-trial care

Harm suffered by participants from trial participation is not anticipated. In the event of complaints and concerns, these can be directed towards the research team, and participants will have the relevant contact details.

Care homes in the intervention arm will be informed that they can retain the training package and intervention materials after the end of the feasibility study. The intervention training package and materials will also be made available to the control arm care homes after the end of the study.

### Outcomes

This study does not have a primary outcome measure. The main aim of this study is to determine the feasibility of a multi-centre cluster-randomised trial for the prevention of oral disease in older people in care homes. The feasibility study will provide necessary information to enable selection of the most appropriate primary outcome measure, estimates of treatment effects and other important parameters to plan for a definitive trial. The outcome measures that will be recorded in this study are as follows:

1. Clinical outcomes (assessed at baseline and 12 months by a dental examiner) will include the number of teeth, number of teeth with coronal and root caries lesions, the proportion of teeth with visible plaque and the proportion of teeth that bleed on probing;
2. Oral symptoms and urgent dental care refers to the number of reported episodes of dental pain, sepsis, discomfort and urgent dental care appointments (collected weekly by care home staff and at baseline, 6 and 12 months by researchers);
3. Health-related quality of life using the EuroQol five dimensions questionnaire – EQ-5D5L [27] (collected at baseline, 6 and 12 months);
4. Oral health-related quality of life using the Oral Impacts on Daily Performances (OIDP) [28] (collected at baseline, 6 and 12 months); and
5. Oral health needs assessed by OHAT [26] (collected by dental examiners at baseline and 12 months).

The EQ-5D is the most commonly used outcome in health economics evaluation studies to calculate QALYs, preferred also by NICE [29]. Its responsiveness has not been proven for oral conditions and therefore the additional use of condition-specific measures is recommended [30]. The OIDP is a brief and widely used oral health-related quality of life outcome measure, validated also amongst older adults in the UK [28]. It is included in the national dental health surveys of adults in the UK [31] and previously used in care homes in London in an interviewer-administered format to provide self-reports of residents about the impact of oral conditions on key aspects of their daily life, such as eating, speaking, cleaning teeth, smiling, relaxing, and enjoying the contact of other people [2].

Data will be collected to facilitate the assessment of the fidelity of the intervention. This refers only to the care homes in the intervention arm of the study and will include information on completion rates (fully, partially or not completed) for the following: (1) OHAT administered by trained care staff to participants; (2) Personal Care Plan for participants; (3) Weekly Oral Hygiene Record of participants; (4) Care Staff Completed Training.

As part of the process evaluation, semi-structured interviews with care home staff (managers, carers and other staff) and residents will assess the intervention’s feasibility (issues relating to recruitment, retention and fidelity) and acceptability. How the intervention could be embedded in standard practice will also be explored using the different domains of Pfadenhauer et al.’s [21] framework (Table 1). Specifically, the framework will be used to determine the factors that are important for implementation.

**Table 1:** Domains and questions for reflection from Pfadenhauer et al. [21]

Main	Questions for reflection
Intervention characteristics	Which intervention characteristics interact with the setting, the context and the implementation? How do these intervention characteristics interact with the setting, the context and the implementation?
Context	How do aspects of the context interact with the intervention? Which aspects of the context interact with the implementation of the intervention?
Implementation theory	Which theoretical underpinning guides the implementation? How does this theory interact with the setting and the context? How does it interact with the intervention?
Implementation process	Which stages of the implementation process are passed through during implementation? How does the implementation process interact with the setting and the context? How does it interact with the intervention?
Implementation strategy	Which strategies are employed during implementation? How do these implementation strategies interact with the setting and the context? How do they interact with the intervention?
Implementation agents	Which agents are involved in the implementation effort? How do these implementation agents interact with the setting and context? How do they interact with the intervention?
Implementation outcomes	Which implementation outcomes are reported with the setting and the context? How do these implementation outcomes interact with the intervention outcomes?
Setting	Which aspects of the setting interact with the intervention? How does the setting interact with the intervention? How does it interact with the context? How does it interact with implementation?

Additional semi-structured interviews with key stakeholders (care home staff and residents, family members and policymakers) will help identify a core set of relevant outcomes, and will also explore issues associated with the valid, reliable and efficient collection and reporting of a core outcome set. During the interview, stakeholders will be presented with information on a range of costs, outcomes and cost per outcome. They will be asked to consider how important these costs and outcomes are and why. A cost-consequence model to inform a future larger scale definitive trial will be produced.

### Participant timeline

**Fig. 2:**
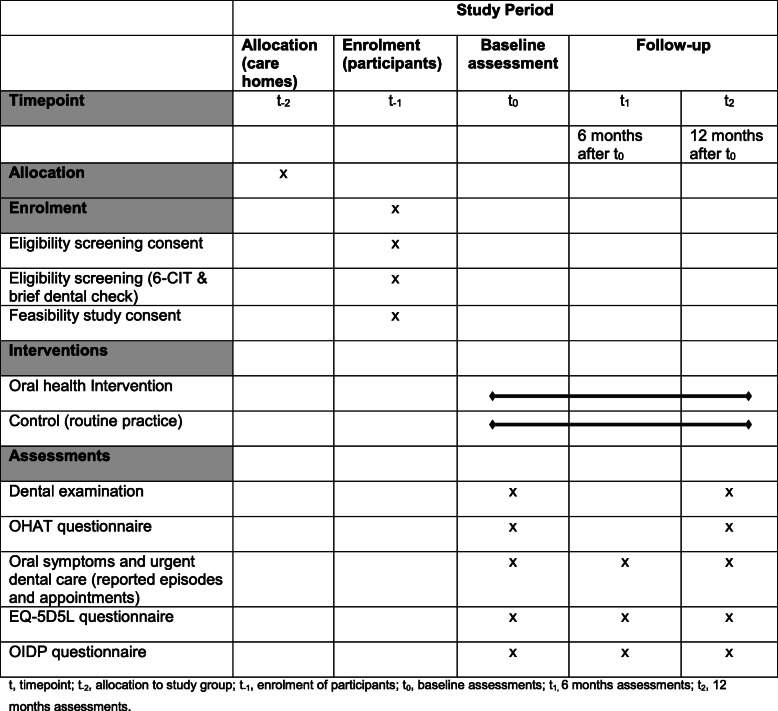
Time schedule of enrolment, interventions, assessments, and visits for participants. t, timepoint; t_−2_, allocation to study group; t_−1_, enrolment of participants; t_0_, baseline assessments; t_1_, 6-month assessments; t_2_, 12-month assessments

Figure 2 provides an overview of the feasibility study time schedule. At the allocation time point (t_−2_), recruited care homes will be randomly allocated to an intervention or control arm. At the enrollment time point (t_−1_), residents within the recruited care homes who are interested in taking part in the study will be asked to provide written consent to undergo two tests to determine their eligibility. If eligible, they will then be asked to provide informed written consent to take part in the feasibility study. The care homes allocated to the intervention arm will be asked to implement the oral health intervention based on the NICE guideline NG48 from baseline (t_0_) to the 12-month time point (t_2_), and the care homes allocated to the control arm will be asked to continue with their usual routine practice during the same 12-month period. Assessments (intervention and control group) will be undertaken at baseline (t_0_), 6 months after baseline (t_1_) and 12 months after baseline (t_2_). All assessments will take place at the care homes. More detailed information about the above processes is provided in the relevant sections below.

As part of the process evaluation aspect of this study, semi-structured interviews with care home staff and residents will be conducted in parallel to the main trial. Similarly, interviews with key stakeholders will also be undertaken in order to develop a cost-consequence model for the project.

### Sample size

The feasibility study will be conducted in approximately 12 care homes (six interventions and six controls) equally divided between the two settings (North London and Northern Ireland). The aim is to recruit 10 residents per home (a minimum of five and a maximum of 20), resulting in an estimated recruited sample of 120 residents. This sample will allow us to establish the feasibility, rates of recruitment and retention and any delivery issues with the proposed intervention and the research methods. Based on NIHR guidance [32], a sample of 120 participants will allow for an estimated attrition rate of 20% to within a 95% confidence interval of +/− 7%.

The semi-structured interviews with care home staff and residents, and other key stakeholders (process evaluation and cost-consequence model, respectively) will be undertaken until saturation of content is reached.

### Recruitment

#### Recruitment for feasibility study

Recruitment will be a two-stage process. The first stage will be the recruitment of the care homes. The research team will make contact with independent care home providers in North London and Northern Ireland to ensure that a broad range of care homes are recruited. The feasibility study will be conducted in 12 care homes (with expected 50% recruitment rate, 24 homes will be approached).

Eligible care homes will be informed about the study through the standard communication routes (letter/email/phone call or in person) and in collaboration with the Clinical Research Networks in London (North Thames and North West London), and with the South Eastern Health and Social Care Trust in Northern Ireland. The ENRICH network (https://enrich.nihr.ac.uk/pages/research-ready-care-home-network) will also be utilised to assist with recruitment. If eligible care homes want further information, a member of the research team will arrange a visit in person to provide the care home managers with an information sheet, further discuss the study and answer any questions about participation. At least 48 hours after discussing the study, eligible care homes will be contacted by the researcher to confirm whether they would like to take part or not.

The second stage will be the recruitment of eligible residents in participating care homes. The aim is to recruit approximately 10 residents per home. A minimum of five and a maximum of 20 participants will be recruited at each care home. For larger care homes, the research team will randomly select a sample of potentially eligible participants. A screening process comprising of three steps will determine eligibility. In step one, the care home manager will identify potentially eligible residents from their personal information stored in the care home records. The care home manager will then pass the names of residents who meet the following criteria to the research assistant: aged 65 years or over, live in the care home full time, not receiving end-of-life care or palliative care, have a good working level of oral English and are currently not taking part in another oral health intervention or programme. Potentially eligible residents will then be given a participant information sheet (PIS) by the care home manager for the two further eligibility tests (as these warrant separate consent). Once consent is obtained (at least 48 h later) for the two further eligibility tests, the research assistant will undertake the 6-CIT test (step two). Residents with normal cognitive function (6-CIT score of 0-7) and those with mild cognitive impairment (6-CIT score of 8-9) will be potentially eligible for inclusion in the study.

In step three, the research assistant will confirm whether the residents are dentate or partially dentate, by performing a brief dental check, known as ‘lift-the-lip’ exercise. If eligible, the research assistant will provide a PIS for the feasibility study and after at least 48 h will ask the resident to complete the informed consent form for the feasibility study (see ‘Who will take informed consent?’ section).

#### Process evaluation—recruitment for semi-structured interviews

In order to assess the intervention’s feasibility and acceptability, a researcher will approach and invite care home staff (managers, carers and others) and residents already enrolled in the study to take part in a semi-structured interview. A purposive sampling frame will be used to ensure a diversity of staff members are recruited. Those interested in taking part will be provided with a PIS. All potential participants will be given at least 48 h from discussing the study with the researcher to decide whether or not they wish to take part.

#### Cost-consequence model—recruitment for semi-structured interviews

Staff (managers, carers and other staff), residents and family members from included care homes will be approached and invited to take part in a semi-structured interview. Policymakers and key stakeholders, such as members of the Health and Social Care Board in Northern Ireland, National Health Service (NHS) Clinical Commissioning Groups, the Care Quality Commission, the Regulation and Quality Improvement Authority (Registration and Inspection Unit in Northern Ireland), PHE and third sector organisations on ageing, will be approached to arrange a meeting or telephone call to discuss the study and provide input.

A purposive sampling frame will be used to ensure a diverse sample is recruited. Those interested in taking part will be provided with a PIS. All potential participants will be given at least 48 h from discussing the study with the researcher to decide whether or not they wish to take part.

### Assignment of interventions: allocation

#### Sequence generation

Eligible care homes will be randomised (via North Wales Organisation for Randomised Trials in Health Clinical Trials Unit, NWORTH CTU) at site level based on a 1:1 ratio (six interventions and six controls). Research assistants at each site will inform the NWORTH CTU when there are two eligible care homes. Care homes will be randomised in pairs, using a dynamic adaptive randomisation algorithm [33]. Care homes will be stratified by geographical location (North London/Northern Ireland).

#### Concealment mechanism

Randomisation will be at care home level. Once care homes have been entered into the system an independent NWORTH member of staff will allocate the homes using a dynamic adaptive randomisation algorithm [33].

#### Implementation

Since this is a cluster feasibility trial, participants will be allocated to the treatment that has been assigned to the care home. An independent NWORTH member of staff will allocate the care homes and a research assistant will enrol participants on to the study.

### Assignment of interventions: Blinding

#### Who will be blinded

Due to the nature of the intervention, the blinding of care homes and residents is not feasible. However, the clinical dental examiners that will record the baseline and outcome measures will be. The care home staff will be instructed not to reveal allocation information to the dental examiners. The study statistician will be blinded to allocation and will be unblinded after primary analysis has been completed.

#### Procedure for unblinding if needed

The study design is open label with only the statistician being blinded. The statistician will be unblinded to allocation only after all the data have been collected, entered into the database, cleaned and primary analysis has been completed.

## Data collection and management

### Plans for assessment and collection of outcomes

#### Feasibility study

All study participants (intervention and control arms) will undergo an oral examination to collect clinical data. Participants will also provide self-report information on person-centred measures via interviewer-administered validated questionnaires and a symptom checklist. The following assessments will take place at the care home for each selected resident:

• Clinical outcomes will include the number of teeth, number of teeth with coronal and root caries lesions, the proportion of teeth with visible plaque and the proportion of teeth that bleed on probing. The dental examination will be undertaken at baseline and at 12 months by trained dental examiners, commissioned by the research team from the South Eastern HSC Trust, in Northern Ireland, and the Whittington Health Dental Services, in North London.

• A brief and practical assessment of the resident’s oral health needs. This will be assessed using the OHAT questionnaire [26]. The dental examiner will undertake this assessment at baseline and 12 months.

• Oral symptoms and urgent dental care: This refers to the number of reported episodes of dental pain, sepsis, discomfort and urgent dental care appointments. Care support staff using a checklist diary log will collect this information weekly. Research assistants will also collect this information at baseline, 6 months and 12 months.

• Health-related quality of life will be assessed through the EQ-5D-5L questionnaire [27]. This questionnaire will be administered by a trained research assistant to all participants at baseline, 6 and 12 months.

• Oral health-related quality of life will be assessed using the OIDP questionnaire [28]. This questionnaire will be administered by a trained research assistant to all participants at baseline, 6 and 12 months.

In addition, data will be collected at care home level (from the care home managers) for all participating care homes. Information will be collected at baseline and at 12 month follow-up and refers to the funding and organisational features of the care home; the number and overall demographic and health characteristics of residents; the number and oral health training of the staff; the provision of oral health programmes; and the arrangements for health care of the residents.

#### Process evaluation

Semi-structured interviews with care home staff and residents will be undertaken to assess the intervention’s feasibility and acceptability, and to explore how the intervention could be embedded in standard practice guided by Pfadenhauer et al.’s framework [21]. The interviews will last between 30 and 60 min and will be digitally recorded. Participants will be provided with the option to have the interview conducted in person at the care home or over the telephone. The interviews will be undertaken in accordance with a protocol consisting of semi-structured open-ended questions.

#### Cost-consequence model

Semi-structured interviews with key stakeholders will be undertaken to identify key outcomes including clinical and quality of life measures, resource use and measures of equity that are likely to inform decision-making. The interviews will be conducted in accordance with a protocol consisting of semi-structured open-ended questions and will last no longer than 20 min. The interviews will be conducted over the phone or in person and will be digitally recorded.

#### COVID-19

The coronavirus (COVID-19) pandemic has impacted excessively on care homes and raises many challenges for their safe operation and protection of the residents and staff. To ensure residents, staff and the research team are protected every precaution will be taken to minimise the risk of infection. Face-to-face visits will only be undertaken where care homes have been COVID-free for at least 14 days. Prior to each face-to-face visit, care homes will be asked to complete a COVID-19 screening questionnaire to assess and minimise any risk. Researchers will also provide a declaration of their own COVID status (no symptoms or contact with known COVID cases for 14 days). During each visit, researchers will wear a face mask, ensure social distancing and perform hand washing/hand sanitization.

### Plans to promote participant retention and complete follow-up

The research team will have regular contact with the recruited care homes throughout the study period. Care homes will be kept well informed of the study’s progress via a quarterly email.

### Data management

Data will be collected using paper Case Report Forms (CRFs), and then transcribed onto a web-based CRF that will not include the participant’s name or other identifiable information that could identify them. Audio recordings of the interviews will be destroyed after verbatim transcripts have been prepared. All data will be stored on a secure dedicated web server. Access will be restricted by user identifiers and passwords (encrypted using a one-way encryption method). All electronic databases will use a participant identification number rather than the participant’s name. Hard copies of data sheets linking the participant identification number to the person’s contact details will be kept securely in a locked filing cabinet in a locked office and will only be accessible to a small number of people who are involved in the study. A more detailed Data Management Plan that complies with the NWORTH’s Standard Operating Procedures addresses details about the data flow and storage, system validation, data cleaning, freezing and locking and sharing and archiving.

### Confidentiality

All data will be stored securely on password protected PCs/laptops and any paper records stored in locked drawers/filing cabinets in secure buildings. All participants’ personal information will be coded and anonymised as far as possible. Only personal identifiers that are essential will be kept and stored securely. Participants’ names will not appear on any documentation associated with the study apart from the Informed Consent Forms and participant contact details, which will be kept in locked filing cabinets separate to any study data. Participants will be allocated a unique study participant identification number, which will be used in any documentation associated with the study. All data will be collected, stored and disseminated in accordance with the General Data Protection Regulation 2018, and policies at the lead universities (University College London, Queen’s University Belfast and Bangor University).

### Plans for collection, laboratory evaluation and storage of biological specimens for genetic or molecular analysis in this trial/future use

Not applicable as no biological specimens will be collected as part of this trial.

## Statistical methods

### Statistical methods for primary and secondary outcomes

As this is a feasibility study, statistical analysis will be restricted to generating summary statistics and confidence intervals. The sensitivity and distribution of the outcome measures proposed for the definitive study will be explored. Recruitment and retention outcomes with associated estimates of precision will be summarised. Acceptability of the interventions and outcome measures, clinical indices (including episodes of pain and hospital admissions) and subjective outcomes by study arm will also be summarised and 95% confidence intervals calculated for the difference in means or proportions as appropriate. All statistical analysis will be undertaken on an intention to treat basis taking into account the clustering of participants within care homes. All statistical analyses will be undertaken at NWORTH CTU. A full Statistical Analysis Plan will be written and agreed by the trial team prior to the completion of data collection. This will be made available for comment by the independent committees.

### Interim analyses

There are no interim analyses planned for this trial. Harm suffered by participants from trial participation is not expected; therefore, this trial has no formal stopping guidelines.

### Methods for additional analyses (e.g. subgroup analyses)

All interviews (process evaluation and cost-consequence model) will be audio-recorded and transcribed verbatim. A thematic analysis, as outlined by Braun and Clarke [34], will be undertaken and a quality checklist will guide analysis and writing [35].

### Methods in analysis to handle protocol non-adherence and any statistical methods to handle missing data

As this is a feasibility study, there will be no imputing of missing data. There will be descriptive statistics produced to describe the amount of missing data for each of the collected outcome measures. This will be used as an indicator of the appropriateness of these measures to be used in a full RCT.

### Plans to give access to the full protocol, participant level-data and statistical code

The Statistical Analysis Plan, data and code can be shared upon reasonable justified request.

## Oversight and monitoring

### Composition of the coordinating centre and trial steering committee

The research team as a whole will meet every 6 months to check progress and decide on operational issues around the project, whilst an Advisory Committee consisting of key research team members will oversee the collaboration between the different partners.

The oversight to the project will be provided through the Study Steering Committee (SSC) and the Data Monitoring and Ethics Committee (DMEC). Both these committees have been appointed. Their roles and responsibilities are determined by the relevant guidance provided by the National Institute for Health Research Evaluation, Trials and Studies Coordinating Centre, and they also comply with the respective requirements for independence.

The SSC consists of a range of national and international experts on different aspects of the project, and Patient and Public Involvement (PPI) representatives. Their expertise collectively covers the fields of gerodontology, dental public health, ageing, interdisciplinary care, nursing, health services research, dementia, clinical trials, medical statistics, epidemiology, operational research, care homes interventions, care homes regulation and policy around ageing. The SSC will have overall responsibility for overseeing the study, ensuring that the trial is conducted in accordance with the principles of Good Clinical Practice and the relevant regulations. The SSC will therefore, provide advice on all aspects of the study, including the project’s continuation or termination.

### Composition of the data monitoring committee, its role and reporting structure

The DMEC will monitor the data and ethical aspects of the study and provide advice on changes to the conduct of the study via recommendations to the SSC. It consists of three independent members that collectively have expertise on dental public health, statistics, health services research and ageing. The DMEC charter will be made available upon reasonable request.

### Adverse event reporting and harms

The adverse events (AEs) reporting period for this study begins as soon as the participant consents to be in the study and ends 1 month after their final data collection. Adverse event data will be collected and recorded on the AEs and serious adverse events (SAEs) CRFs by the research assistant on a monthly basis. Only details of any SAEs that are related to taking part in the study will be reported to the Research Ethics Committee. The occurrence of AEs during the trial will be monitored by the DMEC and SSC.

### Frequency and plans for auditing trial conduct

The sponsor may monitor and conduct audits as per their procedures. Within NWORTH CTU, the TOPIC study will be subject to internal audits on their processes, where applicable.

### Plans for communicating important protocol amendments to relevant parties (e.g. trial participants, ethical committees)

Any modifications to the protocol will be communicated to all relevant parties including the funder, the sponsor, the ethics committee and other relevant authorities. The trial registry entry will be updated and all research sites will receive a revised copy to store in their Investigator site file.

## Dissemination plans

A multifaceted approach will be used to promote the dissemination of the results of this research. The study protocol and also the key findings will be disseminated to the scientific community through conference presentations and peer-reviewed publications. Furthermore, informal dissemination networks will be established via the PPI and stakeholder groups and the developed relationships will be utilised. This will ensure dissemination of information directly to older people, carers and care home managers. New and novel methods to support this dissemination will be developed with the PPI group and they will also create public-friendly summaries of the research. At a service level, formal links with dental commissioners, Consultants in Dental Public Health and Gerodontologists in the UK will be established through the British Association for the Study of Community Dentistry network and the British Society of Gerodontology. Links to The European College of Gerodontology (ECG) and the International Association of Dental Research (IADR) Geriatric Oral Research Group will be utilised. The research team also has strong links with the Council of European Chief Dental Officers (CECDO) and the Platform for Better Oral Health in Europe (PBOHE), a joint initiative of the scientific oral health societies across Europe, with a mission to promote oral health and the cost-effective prevention of oral diseases. This organisation also has links to key stakeholders across Europe.

Furthermore, links with the Centre for Ageing and Dementia, the Centre for Policy on Ageing, the Age Sector Platform and with Age Cymru will complement and enrich the PPI group input and provide a robust channel for dissemination and knowledge transfer to both dependent older people and important policymakers and stakeholders. The research team’s links with PHE, the British Dental Association and the Regulation and Quality Improvement Authority in Northern Ireland will further develop the pathways to impact of this project and promote engagement of all relevant stakeholders. A copy of the findings will also be sent to the commissioner for older people for Northern Ireland.

## Discussion

Care home residents’ oral health is much worse than their peers living in the community. The NICE NG48 guideline provides recommendations for promoting oral hygiene and preventing oral diseases in older people in care homes. Although instructions as to how to implement the NG48 guideline in care homes are provided by NICE, the suggestions do not contain specific, tangible actions required by care home staff to effectively implement the guideline. A complex oral health intervention that is based on the NICE NG48 guideline has therefore been developed as part of this project using a co-design process that was based on working with residents and care home staff. The co-design methods used to refine this complex intervention will maximise its clinical and cultural acceptability through increased understanding of the context and mechanisms for delivery.

This protocol paper describes the process that will be used to assess the feasibility of the complex oral health intervention based on the NG48 guideline. Application of the progression criteria will help evaluate the likely success of a full-scale definitive trial and if modifications to the oral health intervention are required. Furthermore, the parallel process evaluation will provide a valuable insight into how the oral health intervention could be embedded in standard practice. Ultimately, the study will strengthen the evidence base regarding the provision of high-quality oral health care services for ageing populations in care homes.

## Trial status

The study protocol is version 6 (14th April 2020). The COVID-19 pandemic has impacted excessively on care homes and raises many challenges for their safe operation and protection of the residents and staff. This had led to the closing of care homes to all visits in order to mitigate the risk to their vulnerable older residents. As such, recruitment for this study has been postponed due to the COVID-19 pandemic and will commence once access restrictions have been lifted in care homes, and when it is deemed safe to proceed with research. All enhanced cross infection protocols in the care homes will be adhered to.

## Data Availability

At the end of the study, NWORTH CTU will release a data pack containing the raw data extracted from MACRO database, the analysis data sets and any agreed syntax. The Statistical Analysis Plan, data and code can be shared publicly upon reasonable justified request.

## Abbreviations

AE: Adverse event
CRF: Case Report Form
CTU: Clinical trials unit
DMEC: Data Monitoring and Ethics Committee
EQ-5D5L: EuroQol five dimensions questionnaire
HSC: Health and Social Care
NHS: National Health Service
NICE: National Institute for Health and Care Excellence
NIHR: National Institute of Health Research
NWORTH: North Wales Organisation for Randomised Trials in Health
OHAT: Oral Health Assessment Tool
OIDP: Oral Impacts on Daily Performances
PHE: Public Health England
PIS: Participant information sheet
PPI: Patient and Public Involvement
PSP: Priority Setting Partnership
RCT: Randomised controlled trial
SAE: Serious adverse event
SSC: Study Steering Committee
TIDieR: Template for Intervention Description and Replication
UK: United Kingdom
6-CIT: 6-Cognitive Impairment Tool
